# Variations of Serum CRP Levels in Periodontal Health and Diseases: A Clinico-Biochemical Study

**DOI:** 10.3390/diagnostics13152483

**Published:** 2023-07-26

**Authors:** Sidharth Shankar, Shiva Manjunath, Saad Mohammad Alqahtani, Kiran Kumar Ganji, Raghavendra Reddy Nagate, Shankar T. Ghokale, Anil Kumar Nagarajappa, Mukhatar Ahmed Javali, Shreyas Tikare, Mohasin Abdul Khader

**Affiliations:** 1 ECHS, Air Force Hospital, Gorakhpur 273014, India; sidharth.shankar7@gmail.com; 2 Department of Periodontics, Institute of Dental Sciences, Bareilly 243006, India; drmanju75@rediffmail.com; 3 Department of Periodontics and Community Dental Sciences, College of Dentistry, King Khalid University, Abha 61421, Saudi Arabia; s.alqahtani@kku.edu.sa (S.M.A.); rnagati@kku.edu.sa (R.R.N.); jmahmad@kku.edu.sa (M.A.J.); tikane@kku.edu.sa (S.T.); mabdulqader@kku.edu.sa (M.A.K.); 4 Department of Preventive Dentistry, College of Dentistry, Jouf University, Sakaka 72345, Saudi Arabia; 5 Department of Periodontics & Implantology, Datta Meghe Institute of Higher Education & Research, Sharad Pawar Dental College, Sawangi (Meghe), Wardha 442107, India; 6 Department of Oral & Maxillofacial Surgery & Diagnostic Sciences, College of Dentistry, Jouf University, Sakaka 72345, Saudi Arabia; dr.anil.kumar@jodent.org

**Keywords:** acute-phase proteins, C-reactive protein, periodontitis

## Abstract

This study aimed to quantify serum C-reactive protein (CRP) values in periodontally healthy people and explore the relationship between serum CRP levels and chronic periodontitis, and the influence of scaling as well as root planing (SRP) on serum CRP levels. The study included 100 systemically healthy adults (*n* = 100; 50 males and 50 females) who were separated into two groups: Group A (control) *n* = 50; periodontally healthy subjects and Group B (test) *n* = 50; subjects with chronic periodontitis. The test group (group B) was further separated randomly into two groups: B1 (*n* = 25) and B2 (*n* = 25). The clinical parameters and serum CRP levels were measured only once in Group A and before SRP in Group B1 subjects. In Group B2 subjects the clinical parameters and serum CRP levels were measured only after two months following SRP. For group A, B1, and B2 (the readings recorded after SRP) the mean gingival index scores were 0.146, 2.437, and 1.052, respectively, while the plaque index was 0.414, 2.499, and 0.954, respectively. Probing pocket depth (PPD) and clinical attachment loss (CAL) showed statistically significant differences between three groups, with higher values in patients with periodontitis before intervention (2.196 ± 0.49; 1.490 ± 0.23), respectively. Healthy controls (Group A) had a C-reactive protein level of 0.04820 mg/dL, while group B1 (test) had 1.678 mg/dL and 0.8892 mg/dL (group B2). C-reactive protein levels were observed to be greater in the test group (groups B1 and B2), and these differences were statistically significant (*p* < 0.001). Chronic periodontitis enhances blood levels of systemic inflammatory markers like CRP, which has been reduced by periodontal treatment with SRP.

## 1. Introduction

The progressive destruction of periodontal tissues is the result of a chronic inflammatory disease triggered by a dysbiotic dental biofilm [1]. It is important to recognize that bacteria and their products gradually affect the periodontium’s integrity, which may lead to a local inflammatory response as well as a systemic response [2,3]. Inflammatory and immunologic mediators induce an acute phase response in reaction to microbial, viral, or parasitic tissue insults, mechanical or thermal trauma, ischaemic necrosis, or malignant growth [4]. In addition to elevated pro-inflammatory mediator levels, patients with periodontitis exhibit distinct haematological alterations, including elevated C-reactive protein (CRP) levels [4,5,6].

As a result of pro-inflammatory cytokines released by hepatocytes during inflammatory/infectious processes, C-reactive protein is synthesized. There are two forms of CRP, native pentameric CRP and monomeric CRP, which bind to different receptors and lipid rafts and exhibit different functions [7]. Several large-scale, prospective studies have shown that CRP is also associated with chronic inflammation, although it is known as a biomarker of acute inflammation [8,9,10]. Cytokines are released as a result of tissue injury, resulting in acute phase reactions, and causing the liver to generate acute phase proteins such as CRP. During acute phase reactions, plasma CRP rises dramatically [11]. In vitro, CRP displays both anti-inflammatory and pro-inflammatory effects. Furthermore, the pro-inflammatory effects encompass ligand-bound CRP’s ability to stimulate the complement system [12].

CRP is normally present in trace amounts in the plasma (0.6 mg/dL) in healthy people, and its concentration rises in the blood in response to inflammation or tissue destruction. These levels have been found to return to normal after the inflammation, or tissue destruction resolves [7,13]. Women with CRP levels of 3 mg/L or above have a greater risk of hypertension, according to research, and this risk is independent of other risk factors such as obesity, hyperlipidemia, or diabetes [14,15]. In other studies, increased CRP levels have been put forth to indicate mild inflammation associated with atherosclerotic vascular disease [16,17].

Elevated serum CRP levels are caused by active tissue-damaging processes and indicate current disease activity. It is also considered a diagnostic adjunct in managing systemic infection [18]. Periodontitis is a slowly progressive and destructive disease with periods of exacerbation and remission. Serum CRP levels in patients with periodontal disease are higher, according to current findings based on systematic review [19]. This suggests a relationship between destructive periodontal disease and an increased risk of atherosclerotic and cardiovascular problems [20]. The exact method by which CRP contributes to cardiovascular disease is unknown. CRP, on the other hand, may stimulate the complementary system and play a role in the production of foam cells [14,21]. According to research, elevated CRP levels associated with other clinical diseases may serve as an extra stimulus for a systemic inflammatory response [22,23]. Serum CRP measurement in clinical settings could be used to screen for organic illnesses. In some inflammatory, infective, and ischemic disorders, such as periodontal disease, serum CRP has also been used as a measure of disease activity and response to therapy [24,25]. Therefore, the null hypothesis for this study stated that the periodontal therapy in chronic periodontitis doesn’t influence the CRP levels. As a result, the goal of this study was to compare serum CRP levels and clinical parameters of patients with chronic periodontitis before and after scaling and root planing (SRP).

## 2. Materials and Methods

This study was performed in 2015 at the Institute of Dental Sciences in Bareilly, Uttar Pradesh, India. The subjects who had periodontal treatment in the last six months, were on antibiotics for the previous three months, had a history of smoking, tobacco chewing, or alcohol abuse, pregnant and lactating patients, and patients on steroid therapy were excluded from the study. Written informed consent was obtained from the participants prior to enrollment.

### 2.1. Study Design

**Figure 1:**
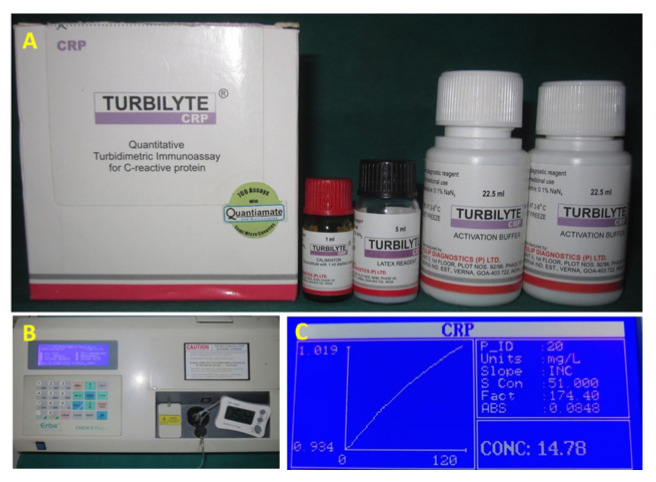
(**A**) Turbilyte CRP reagent, (**B**) Spectrophotometer, (**C**) CRP concentration graph.

**Figure 2:**
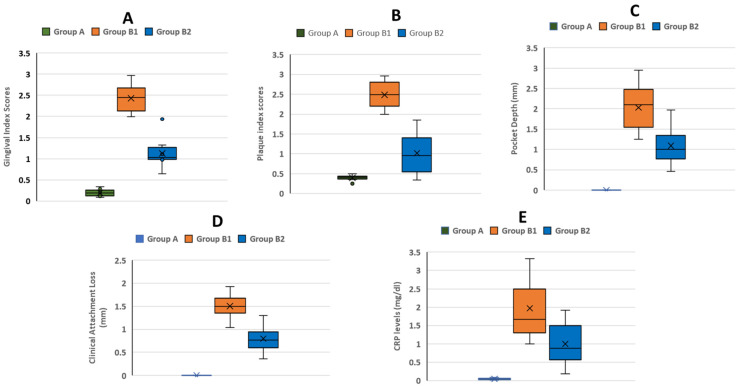
(**A**) Mean gingival index scores, (**B**) mean plaque index scores, (**C**) mean probing pocket depth scores, (**D**) mean clinical attachment level scores, (**E**) mean C-Reactive Protein levels. The “x” means outlier.

The sample size for the study was estimated using G-Power computing tool. We calculated the effect size (d = 0.5784) using a standardized effect size, with a power (priori power analysis) set to 0.80 that would be used when combining the results of the study with the results of other studies, such as in meta-analysis [26]. Earlier studies found a minimally significant difference (MID) of 0.50 (clinical significance) in scores. The systemic status of the participants was assessed by personal interview and confirmed by clinical assessment. The study included 100 systemically healthy adults (*n* = 100; 50 males and 50 females) whose age ranged between 30–55 years. Participants were randomized into two groups: Group A (control group: Figure 1) *n* = 50; periodontally healthy subjects and Group B (test) *n* = 50; subjects with chronic periodontitis. The test group (group B: Figure 2) was further separated randomly into two groups: B1 (*n* = 25) and B2 (*n* = 25). The clinical parameters and serum CRP levels were measured only once in Group A and before SRP in Group B1 subjects. In Group B2 subjects the clinical parameters and serum CRP levels were measured only after two months following SRP (as the same set of participants were enrolled in both Group B1 and Group B2). Orthopantomogram was taken to confirm the bone loss to aid in initial diagnosis. The clinical parameters recorded included gingival Index (GI); Loe and Silness [27], plaque index (PI); Silness and Loe [28], probing pocket depth (PPD) and clinical attachment level (CAL), and C-reactive protein (CRP) levels.

### 2.2. Methodology and Sample Collection

After taking a comprehensive case history, the clinical parameters (GI, PI, PPD, CAL, and CRP) were measured and assessed by the authors as baseline data. After that, 2 mL of blood was drawn for all the subjects from the antecubital fossa by venipuncture under the aseptic condition to estimate baseline serum CRP levels. The blood sample was then centrifuged (C-95416, Remi Elecktrotechnik Ltd., Mumbai, India) at 3000 r.p.m. (revolutions per minute) for 10 min, and then the serum was separated. The serum sample was stored in the refrigerator (2–8 °C) until transported in an ice bag to the lab, where the CRP level was analyzed by the immunoturbidity method. Subsequently, in Group B1 and Group B2, supra- and subgingival ultrasonic scaling was performed at the initial visit, and after 7–10 days of time thorough area-by-area root planing was performed using area-specific Gracey curettes (Hu-Friedy^®^., Chicago, IL, USA). Oral hygiene instructions were given and reinforced at every regular recall interval visit during the maintenance phase. All of the above clinical and biochemical parameters were repeated in Group B2 subjects two months after following SRP [19].

### 2.3. Laboratory Procedure for Assessment of C-Reactive Protein Levels in Serum

C-reactive protein levels were determined using quantitative Turbidimetric Immunoassay Turbilyte CRP (B68959, Tulip Diagnostics Ltd., Panaji, India) (Figure 1A) [29]. An activation buffer was added to the test specimen. After that, the Turbilyte CRP reagent was introduced and allowed to react. The creation of an insoluble compound triggered the presence of CRP in the test specimen responsible for the turbid appearance was determined by a spectrophotometer (Chem 5-Plus V2, Transasia Bio-medicals Ltd., Mumbai, India) at 546 nm wavelength (Figure 1B). The increase in turbidity suggests that the specimen being tested has high levels of CRP.

### 2.4. Test Procedure

The reagents and the samples were brought to room temperature before being reconstituted exactly with 1.0 mL of distilled water using Turbilyte CRP calibrator. After 10 min, the vial was gently swirled until the solution attained homogeneity. Once reconstituted, it became ready to use for the preparation of the CRP calibration curve. We mixed 450 µL of Turbilyte CRP activation buffer and 50 µL of diluted calibrator well and incubated this for five minutes at 37 °C. Absorbance (Al) was noted. Then, 50 µL of Turbilyte CRP reagent (preincubated at 37 °C) was added and mixed gently. The absorbance (A2) was noted at the end of precisely five minutes.

### 2.5. Calculations

Calculation of ΔA (A2 − A1) for the calibrator-A graph of ΔA versus CRP concentration was plotted (Figure 1C). For detecting CRP levels in the test specimen, the above steps were repeated, the test specimen was used instead of calibrator, and ΔA was calculated for the test specimen. Interpolation of ΔA of the diluted test specimen was performed on the calibration curve, and the CRP concentration of the test specimen was obtained. The CRP concentration was multiplied with the test specimen’s dilution factor to obtain CRP concentrations in the neat test specimen.

### 2.6. Statistical Analysis

The statistical analysis was carried out using Statistical Package for Social Sciences (SPSS Inc., Chicago, IL, USA, version 20.0 for Windows). Mean values of the clinical parameter and CRP levels between the groups were analyzed by one-way ANOVA followed by Bonferroni post hoc test for intergroup multiple comparison. This was performed at a significance level of α ≤ 0.05.

## 3. Results

The present study showed that all three groups were different in their gingival and plaque index scores with more clinically severe scores in group B1 (before SRP treatment) compared to group A (healthy individuals) and B2 (after SRP treatment) (*p* < 0.05). Similarly, PPD and CAL showed statistically significant differences between three groups, with higher values in patients with periodontitis before intervention (2.196 ± 0.49; 1.490 ± 0.23), respectively. The mean Gingival Index score for Group A was 0.146, for Group B1 was 2.4372, and for Group B2 was 1.0522. Statistical analysis showed that mean differences between groups were found to be significant (*p* < 0.001). Plaque index for all the groups were compared and it was seen that in Group A the mean score was 0.4142; in Group B1 it was 2.499; and in Group B2 it was 0.9544. A statistically significant mean difference was seen (*p* < 0.001), when mean plaque scores for all the groups were compared. These results show that the plaque index of the patients was poor in Group B1 when compared to Group A and Group B2. For periodontitis patients in Group B1, the mean level of C-reactive protein was 1.678 mg/dL and in Group B2 it was 0.8892 mg/dL after SRP. The mean level of C-reactive protein was higher in Group B1 and Group B2; however, the results were statistically significant (*p* < 0.001). When serum levels of C-reactive protein for controls Group A were compared to those of periodontitis patients in Group B1, the results were also significant (*p* < 0.001) (Table 1) (Figure 2).

The mean CRP level for three groups in descending order were group B1 (1.678 ± 0.58), Group B2 (0.889 ± 0.04) and Group A (0.048 ± 0.02). Groupwise comparison revealed significant difference between all groups and also between untreated chronic periodontitis subjects, who demonstrated higher CRP levels compared to SRP treated chronic periodontitis subjects and healthy subjects (*p* < 0.05) [Table 2].

## 4. Discussion

Over the last two decades, there has been an increased awareness and interest in the influence of oral health on cardiovascular diseases. CRP is a periodontal disease indicator, and it increases by several folds in response to infection or inflammation. In various inflammatory, infectious, and ischemic disorders, it is a measure of disease activity and response to treatment [30]. Several studies have shown the potential to explain instances where the intraoral source of infection has caused systemic inflammatory response despite the absence of other contributing factors, thus posing an increased threat of cardiovascular diseases in healthy patients [22]. CRP is a serum protein synthesized in the liver only during an inflammatory disease and has a half-life of 19 h. The rate of synthesis and secretion increases within hours of an acute injury or onset of inflammation. It may reach as high as 20 times the normal value. An elevated serum concentration of CRP is evidence of active tissue-damaging processes, an indicator of current disease activity, and a diagnostic adjunct in the management of systemic infection. It plays a protective role in recognizing foreign pathogens and initiating their elimination, probably activating the classic pathway of complement through complement activation [17]. Taking into consideration the short half life of 19 h, it would be deemed necessary for the researchers to evaluate the concentration of CRP very frequently; however, this was not pursued in the current investigation. The monitoring period for evaluating the changes in CRP levels was scientifically supported by the conclusion drawn out of the systematic review on CRP and periodontal diseases by Machado et al. [19]. The conclusions presented in this systematic review were that the periodontitis treatment induces a short-term acute inflammatory increase when performed in an intensive session, whilst a progressive reduction in up to 6 months was demonstrated when performed in multiple visits.

Periodontitis is a disease of the periodontium that is characterized by periods of active tissue destruction and periods of relative quiescence. The positive C-reactive protein level is observed during the active tissue destruction period. Patients with levels of CRP below 0.6 mg/dL could be in the quiescence period of periodontitis, which gives a negative finding for in terms of CRP levels. On the contrary, studies found that CRP levels did not vary significantly between healthy and diseased sites [31,32]. This could be because many diseased sites were likely stable and some healthy sites may have been undergoing active attachment loss. Quantitative analysis of CRP levels in the blood can help in assessment and management of inflammatory periodontal disorders, as well as determination of the link between periodontal health and other issues such as cardiovascular disease [24]. The results of the current study suggest that there was increased severity of inflammation and disease activity in Group B1 and Group B2 when compared to Group A. The results indicate that the plaque index of Group B1 patients was significantly higher compared to both Group A and Group B2, suggesting poorer oral hygiene and a greater risk for periodontal disease. However, the comparison of Group A and Group B2 was also found to be statistically significant (*p* < 0.001). This decline in serum CRP levels in Group B2 explains the reduction in periodontal inflammation after SRP. Based on these findings, we rejected the null hypothesis of the study as the higher CRP levels in the test group (Group A) suggested an increased severity of inflammation and disease activity compared to group B1 and group B2. Our findings also showed higher gingival inflammation and disease severity in periodontitis patients than control subjects. The positive CRP level was observed during the active tissue destruction period. The patients with CRP levels below 0.6 mg/dL could be in the quiescence period of periodontitis, which may have resulted in negative findings for the CRP levels. On the contrary, other studies [33] found that the difference in CRP levels between healthy and sick locations was not significant. This could be attributed to the fact that many diseased areas were perhaps stable, and other non-diseased sites may have been undergoing active attachment loss.

Another significant finding of this study is the improvement in all clinical parameters, such as the reduction in probing pocket depth and the increase in clinical attachment, in the test group. As a result, the considerable drop in serum CRP levels in the test group can be linked to the above-mentioned improved clinical indicators. As a result, this study shows that SRP reduces serum CRP levels significantly. This study’s results are in accordance with the other studies [34,35]. The reagent (Turbidimetric Immunoassay Turbilyte CRP) used in the present study had the advantage of being economical. Furthermore, it was possible to get minute values of serum CRP level in all the subjects with the quantitative Autoanalyser (ERBA-CHEM 5 PLUS). This enabled us to compare the normal values of serum CRP of periodontally healthy subjects, i.e., control group A to those of chronic periodontitis of test Group B2 following SRP. The decrease in serum CRP level in Group B2 with improved periodontal condition after SRP and approached the levels of group A control subjects; however, this decline was statistically significant (*p* < 0.001). These findings are comparable to the study results of George et al. [34]. The results of the above study suggested that destructive periodontal diseases contribute to systemic inflammatory response, as indicated by the levels of C-reactive protein in serum. This level in the serum is associated with the degree of inflammation and amount of destruction in the periodontium. Periodontal disease, a common condition, may predispose the affected patient to cardiovascular diseases by increasing the levels of this acute phase protein. Established risk factors for “high-normal” values of CRP within the general population include older age, cigarette smoking, chronic bacterial infections, and chronic bronchial inflammation. However, raised CRP levels have been observed among individuals with no apparent established risk factors for elevated CRP, suggesting that other pathological conditions may constitute an additional stimulus for a systemic inflammatory response among some individuals [19]. As in this current investigation, only stimulus form chronic periodontal infection triggering the systemic inflammatory response has been taken as a confounding factor; however, these results may vary in individuals with variations in age and smoking habits (not assessed in the current study)—a of the limitation for the current study. Furthermore, the results of the above study may be subjected to several limitations, like the small sample size and short follow up. Thus, long term interventional studies with larger sample size are needed to further evaluate the role of periodontal diseases in these conditions.

## 5. Conclusions

Within the limitations of the current study, it can be suggested that destructive periodontal diseases contribute to systemic inflammatory response, as indicated by the levels of CRP in serum. However, after scaling and root planing treatment, the serum levels of C-reactive protein decreased significantly, suggesting that this treatment is effective in reducing periodontal inflammation. These findings highlight the importance of timely periodontal treatment in managing periodontitis and reducing CRP levels in affected individuals.

## Figures and Tables

**Table 1:** Comparison of Clinical parameters and CRP levels among three groups.

Clinical Parameter	Group A	Group B1	Group B2	F	*p* Value
Gingival Index (GI)	0.14 ± 0.03	2.43 ± 0.33	1.05 ± 0.34	853.27	0.00 *
Plaque index (PI)	0.41 ± 0.07	2.49 ± 0.27	0.95 ± 0.37	749.49	0.00 *
Probing pocket depth (PPD)	0.00 ± 0.00	2.19 ± 0.49	1.20 ± 0.40	443.92	0.00 *
Clinical attachment Loss (CAL)	0.00 ± 0.00	1.49 ± 0.23	0.77 ± 0.21	840.56	0.00 *
C–Reactive Protein (CRP)	0.04 ± 0.02	1.67 ± 0.58	0.88 ± 0.04	216.73	0.00 *

* *p* value < 0.05 statistically significant.

**Table 2:** Post hoc analysis of clinical parameters and CRP levels among the three groups.

Parameter	Group		Mean Difference	Std. Error	95% Confidence Interval		
					Lower Bound	Upper Bound	*p* Value
GI	A	B1	−2.29	0.06	−2.43	−2.16	0.00 *
		B2	−0.91	0.06	−1.04	−0.77	0.00 *
	B1	A	2.29	0.06	2.16	2.43	0.00 *
		B2	1.39	0.06	1.25	1.52	0.00 *
	B2	A	0.91	0.06	0.77	1.04	0.00 *
		B1	−1.39	0.06	−1.52	−1.25	0.00 *
PI	A	B1	−2.08	0.06	−2.22	−1.95	0.00 *
		B2	−0.56	0.06	−0.69	−0.43	0.00 *
	B1	A	2.08	0.06	1.95	2.22	0.00 *
		B2	1.52	0.06	1.39	1.66	0.00 *
	B2	A	0.56	0.06	0.43	0.69	0.00 *
		B1	−1.52	0.06	−1.66	−1.39	0.00 *
PPD	A	B1	−2.20	0.07	−2.37	−2.02	0.00 *
		B2	−1.21	0.07	−1.39	−1.03	0.00 *
	B1	A	2.20	0.07	2.02	2.37	0.00 *
		3	0.99	0.07	0.81	1.17	0.00 *
	B2	A	1.21	0.07	1.03	1.39	0.00 *
		B1	−0.99	0.07	−1.17	−0.81	0.00 *
CAL	A	B1	−1.49	0.04	−1.58	−1.40	0.00 *
		B2	−0.78	0.04	−0.87	−0.69	0.00 *
	B1	A	1.49	0.04	1.40	1.58	0.00 *
		B2	0.71	0.04	0.62	0.80	0.00 *
	B2	A	0.78	0.04	0.69	0.87	0.00 *
		B1	−0.71	0.04	−0.80	−0.62	0.00 *
CRP	A	B1	−1.63	0.08	−1.82	−1.44	0.00 *
		B2	−0.84	0.08	−1.03	−0.65	0.00 *
	B1	A	1.63	0.08	1.44	1.82	0.00 *
		B2	0.79	0.08	0.60	0.98	0.00 *
	B2	A	0.84058 *	0.08	0.65	1.03	0.00 *
		B1	−0.78942 *	0.08	−0.98	−0.60	0.00 *

* *p* value < 0.05 statistically significant.

## Data Availability

Data are contained within the article.

## References

[1] Bowen W.H., Burne R.A., Wu H., Koo H. (2018). Oral biofilms: Pathogens, matrix, and polymicrobial interactions in microenvironments. Trends Microbiol..

[2] Hajishengallis G., Chavakis T. (2021). Local and systemic mechanisms linking periodontal disease and inflammatory comorbidities. Nat. Rev. Immunol..

[3] Hajishengallis G. (2015). Periodontitis: From microbial immune subversion to systemic inflammation. Nat. Rev. Immunol..

[4] Ebersole J.L., Dawson D., Emecen-Huja P., Nagarajan R., Howard K., Grady M.E., Thompson K., Peyyala R., Al-Attar A., Lethbridge K. (2017). The periodontal war: Microbes and immunity. Periodontology 2000.

[5] Botelho M., Gao X., Jagannathan N. (2019). A qualitative analysis of students’ perceptions of videos to support learning in a psychomotor skills course. Eur. J. Dent. Educ..

[6] Paraskevas S., Huizinga J.D., Loos B.G. (2008). A systematic review and meta-analyses on C-reactive protein in relation to periodontitis. J. Clin. Periodontol..

[7] Pepys M.B., Hirschfield G.M. (2003). C-reactive protein: A critical update. J. Clin. Investig..

[8] Cardoso F.S., Ricardo L.B., Oliveira A.M., Horta D.V., Papoila A.L., Deus J.R., Canena J. (2015). C-reactive protein at 24 hours after hospital admission may have relevant prognostic accuracy in acute pancreatitis: A retrospective cohort study. GE Port. J. Gastroenterol..

[9] Hu L., Shi Q., Shi M., Liu R., Wang C. (2017). Diagnostic value of PCT and CRP for detecting serious bacterial infections in patients with fever of unknown origin: A systematic review and meta-analysis. Appl. Immunohistochem. Mol. Morphol..

[10] Memar M.Y., Alizadeh N., Varshochi M., Kafil H.S. (2019). Immunologic biomarkers for diagnostic of early-onset neonatal sepsis. J. Matern.-Fetal Neonatal Med..

[11] Jain S., Gautam V., Naseem S. (2011). Acute-phase proteins: As diagnostic tool. J. Pharm. Bioallied Sci..

[12] Lagrand W.K., Visser C.A., Hermens W.T., Niessen H.W., Verheugt F.W., Wolbink G.-J., Hack C.E. (1999). C-reactive protein as a cardiovascular risk factor: More than an epiphenomenon?. Circulation.

[13] Loos B.G. (2005). Systemic markers of inflammation in periodontitis. J. Periodontol..

[14] Aoyama N., Suzuki J.-i., Kobayashi N., Hanatani T., Ashigaki N., Yoshida A., Shiheido Y., Sato H., Izumi Y., Isobe M. (2018). Increased oral porphyromonas gingivalis prevalence in cardiovascular patients with uncontrolled diabetes mellitus. Int. Heart J..

[15] Sesso HBuring J.E., Rifai N., Blake G.J., Gaziano J.M., Ridker P.M. (2003). C-reactive protein and the risk of developing hypertension. JAMA.

[16] Bolla V., Kumari P.S., Munnangi S.R., Kumar D.S., Durgabai Y., Koppolu P. (2017). Evaluation of serum c-reactive protein levels in subjects with aggressive and chronic periodontitis in comparison with healthy controls: A clinico-biochemical study. Int. J. Appl. Basic Med. Res..

[17] Marnell L., Mold C., Du Clos T.W. (2005). C-reactive protein: Ligands, receptors and role in inflammation. Clin. Immunol..

[18] Chait A., Han C.Y., Oram J.F., Heinecke J.W. (2005). Thematic review series: The immune system and atherogenesis. Lipoprotein-associated inflammatory proteins: Markers or mediators of cardiovascular disease?. J. Lipid Res..

[19] Machado V., Botelho J., Escalda C., Hussain S.B., Luthra S., Mascarenhas P., Orlandi M., Mendes J.J., D’Aiuto F. (2021). Serum C-reactive protein and periodontitis: A systematic review and meta-analysis. Front. Immunol..

[20] Carrizales-Sepúlveda E.F., Ordaz-Farías A., Vera-Pineda R., Flores-Ramírez R. (2018). Periodontal disease, systemic inflammation and the risk of cardiovascular disease. Heart Lung Circ..

[21] Wu T., Trevisan M., Genco R.J., Falkner K.L., Dorn J.P., Sempos C.T. (2000). Examination of the relation between periodontal health status and cardiovascular risk factors: Serum total and high density lipoprotein cholesterol, C-reactive protein, and plasma fibrinogen. Am. J. Epidemiol..

[22] Kim H.-J., Cha G.S., Kim H.-J., Kwon E.-Y., Lee J.-Y., Choi J., Joo J.-Y. (2018). Porphyromonas gingivalis accelerates atherosclerosis through oxidation of high-density lipoprotein. J. Periodontal Implant. Sci..

[23] Slade G., Offenbacher S., Beck J., Heiss G., Pankow J. (2000). Acute-phase inflammatory response to periodontal disease in the US population. J. Dent. Res..

[24] Gupta S., Suri P., Patil P.B., Rajguru J.P., Gupta P., Patel N. (2020). Comparative evaluation of role of hs C-reactive protein as a diagnostic marker in chronic periodontitis patients. J. Fam. Med. Prim. Care.

[25] Swaroop Chandy K.J., Sankaranarayanan A., Issac A., Babu G., Wilson B., Joseph J. (2017). Evaluation of C-reactive protein and fibrinogen in patients with chronic and aggressive periodontitis: A clinico-biochemical study. J. Clin. Diagn. Res. JCDR.

[26] Berben L., Sereika S.M., Engberg S. (2012). Effect size estimation: Methods and examples. Int. J. Nurs. Stud..

[27] Loe H., Silness J. (1963). Periodontal disease in pregnancy (I). Prevalence and severity. Acta Odontol. Scand..

[28] Silness J., Loe H. (1964). Periodontal disease in pregnancy (II). Correlation between oral hygiene and periodontal condition. Acta Odontl. Scand..

[29] Chopra R., Patil S.R., Kalburgi N.B., Mathur S. (2012). Association between alveolar bone loss and serum C-reactive protein levels in aggressive and chronic periodontitis patients. J. Indian Soc. Periodontol..

[30] Sibraa P.D., Reinhardt R.A., Dyer J., DuBois L. (1991). Acute-phase protein detection and quantification in gingival crevicular fluid by direct and indirect immunodot. J. Clin. Periodontol..

[31] Ito H., Numabe Y., Hashimoto S., Sekino S., Murakashi E., Ishiguro H., Sasaki D., Yaegashi T., Takai H., Mezawa M. (2016). Correlation between gingival crevicular fluid hemoglobin content and periodontal clinical parameters. J. Periodontol..

[32] Mehta S., Sahana Selvaganesh D., Thiyaneswaran N. (2023). Evaluation and Comparison of Wound Healing Efficiency in Obese Patients with Elevated C-Reactive Protein Level: An In-Vivo Study. J. Pharm. Negat. Results.

[33] Bansal T., Pandey A., Deepa D., Asthana A.K. (2014). C-reactive protein (CRP) and its association with periodontal disease: A brief review. J. Clin. Diagn. Res. JCDR.

[34] George A.K., Janam P. (2013). The short-term effects of non-surgical periodontal therapy on the circulating levels of interleukin-6 and C-reactive protein in patients with chronic periodontitis. J. Indian Soc. Periodontol..

[35] Kamil W., Al Habashneh R., Khader Y., Al Bayati L., Taani D. (2011). Effects of nonsurgical periodontal therapy on C-reactive protein and serum lipids in Jordanian adults with advanced periodontitis. J. Periodontal. Res..

